# MSC-based therapy in female pelvic floor disorders

**DOI:** 10.1186/s13578-020-00466-4

**Published:** 2020-09-10

**Authors:** Yizhen Sima, Yisong Chen

**Affiliations:** Department of Gynecology, Obstetrics and Gynecology Hospital of Fudan University, 128 Shen Yang Road, Shanghai, 200011 People’s Republic of China

**Keywords:** Mesenchymal stem cells, Mesenchymal stem cells transplantation, Cell- and tissue-based therapy, Pelvic floor disorders

## Abstract

Mesenchymal stem cells (MSCs), also referred to as multipotent stromal cells or mesenchymal stromal cells, are present in multiple tissues and capable of differentiating into diverse cell lineages, holding a great promise in developing cell-based therapy for a wide range of conditions. Pelvic floor disorders (PFDs) is a common degenerative disease in women and may diminish a woman’s quality of life at any age. Since the treatments for this disease are limited by the high rates of recurrence and surgical complications, seeking an ideal therapy in the restoration of pelvic floor function is an urgent issue at present. Herein, we summarize the cell sources of MSCs used for PFDs and discuss the potential mechanisms of MSCs in treating PFDs. Specifically, we also provide a comprehensive review of current preclinical and clinical trials dedicated to investigating MSC-based therapy for PFDs. The novel therapy has presented promising therapeutic effects which include relieving the symptoms of urinary or fecal incontinence, improving the biological properties of implanted meshes and promoting the injured tissue repair. Nevertheless, MSC-based therapies for PFDs are still experimental and the unstated issues on their safety and efficacy should be carefully addressed before their clinical applications.

## Background

Pelvic floor disorders (PFDs) are a group of degenerative conditions that include urinary incontinence (UI), fecal incontinence, pelvic organ prolapse (POP), and other sensory or emptying abnormalities of the lower urinary and gastrointestinal tracts, caused by the weakening of pelvic floor supportive tissues and occurring independently or simultaneously. PFDs have an extremely high prevalence in women, affecting almost 25% of women older than 20 years in the United States, and UI is the most common disorder with a prevalence of 17% in the general population [1]. Stress urinary incontinence (SUI) is the subtype of UI, and about 50% of UI patients are classified as having SUI [2]. Despite being a common disease, the exact etiology and pathogenesis of PFDs remain poorly understood. Many risk factors are related to PFDs [3], including parity, vaginal delivery, age, menopause, chronic cough, obesity, and constipation. These factors may cause abnormal metabolism of extracellular matrix and dysfunction of the pelvic supportive tissues such as cardinal and uterosacral ligaments [4], levator ani muscle [5], and urethral sphincter [6], contributing to the development of PFDs. Although the treatment principles for PFDs vary from different manifestations of the patients, current managements for PFDs can be generally divided into surgical and non-surgical treatments. Non-surgical treatments, including pelvic floor muscle physiotherapy, biofeedback, pessaries, and electrical stimulation [7], have the effects on relieving symptoms and are recommended for the newly diagnosed patients, but they do not offer an anatomy restoration of the pelvic floor. Surgical treatments are recommended for patients who have failed the conservative managements, but surgical treatments are associated with notable complications [8, 9]. The limitations of current managements for PFDs highlight the need to develop new treatments. Restoration of the pelvic floor structures and improvement of the pelvic floor functions through cell therapy has been investigated in many studies. MSCs, as a highly investigated population in regenerative medicine, hold a great promise to enhance tissue repair and have yielded therapeutic effects in a large spectrum of diseases such as graft-versus-host disease (GVHD) [10], cardiac diseases [11] and multiple sclerosis [12]. There is a growing body of literature that recognizes the effectiveness of MSCs-based therapy for treating PFDs both in preclinical experiments and in a small number of clinical trials. Although most of them are preliminary studies, the symptoms of PFDs were relieved both in animal models and human subjects.

This review summarizes the cell sources of MSCs used for PFDs and the roles MSCs playing in the treatment of PFDs, and analyses the recent studies concerning MSC-based therapy for PFDs. It is hoped that this review will contribute to a better understanding of MSCs applications in disease therapy and provide references for the future investigations on MSC-based therapy in PFDs.

### MSCs and MSC-based therapy

Before being named as mesenchymal stem cells, MSCs were first identified from bone marrow and described as colony-forming units-fibroblasts for their fibroblast-like appearance and the ability to form colonies in vitro [13]. In 1990s, Caplan first put forward the term “mesenchymal stem cells” and Friedenstein et al. described the multilineage potential of MSCs that could differentiate into tissues of mesodermal origin such as adipocytes, chondroblasts, and osteoblasts in vitro [14], stimulating the imagination of this intriguing cell type in tissue regeneration. Although MSCs were first isolated from the bone marrow, they have been harvested from many other fetal and adult tissues, including adipose [15], umbilical cord [16], placenta [17], amniotic fluid [18], skin [19], and dental pulp [20]. In 2006, the International Society for Cellular Therapy established minimal criteria to define MSCs derived from multiple tissues and organs, which include the adherent plastic property, surface markers and in vitro multilineage differentiation potential of MSCs [21]. According to that criteria, many preclinical and clinical trials identified MSCs from different origins and applied MSCs into therapeutics. The biological properties such as multilineage potential and immune modulation make MSCs a promising treatment option for a variety of clinical conditions. For example, the phase III trials have been conducted in congestive heart failure [22] and Crohn’s disease [23]. Furthermore, MSCs have secured conditional approval to treat children with GVHD in several countries [24].

Nevertheless, MSC-based therapies have demonstrated excellent therapeutic effects, but there are many unknowns and controversies of MSCs as well as MSC-based therapies. A particular challenge for the field is to set criteria for MSCs. With no specific marker to define MSCs, the surface markers vary between the MSCs derived from different origins [25]. Moreover, the studies identifying and characterizing MSCs are mostly based upon in vitro work, and thus it is hard to qualify the in vivo multilineage potential of MSCs.

Over the last decade, researchers have never stopped to discover the nature of MSCs. Studies revealed that MSCs derived from different origins would exhibit widely different transcriptomic signatures, biological functions, and in vivo developmental potentials [26, 27], which adds new complexities to the identification of MSCs. Besides, there is a growing body of literature recognizes that most MSCs were derived from pericytes [28, 29] and they functionally improved tissue repair or modulated immunity through the paracrine effect rather than differentiation [30–32]. Considering the above evidences challenging the term “mesenchymal stem cells”, and to prevent the abuse of MSCs as a cure-all in business activities, academics and experts recommended to change the term into “medical signaling cells”, reserving the name “MSCs” [33, 34]. Taken together, it is important to further investigate the properties of MSCs as well as to identify the criticisms of their therapeutic uses, because only then can promote their translation from the bench to the bedside.

### Sources of MSCs in treatment of PFDs (Fig. 1)

*Bone marrow*-*derived MSCs (BM*-*MSCs)* are the first discovered MSCs, with the well-studied biological properties, usually considered as the gold standard cell type when investigating the properties of MSCs from other tissues. The previous studies reported BM-MSCs and Adipose-derived MSCs represented the optimal MSCs sources due to their outstanding differentiation capacity [35, 36]. Also, given the immunomodulatory property, BM-MSCs have been used in the treatment of GVHD [10, 37]. However, MSCs are relatively rare in bone marrow (approximately 1 per 10,000 cells) and traditional bone marrow procurement is painful for patients, which may restrict the application of this cell population in PFDs.

**Fig. 1:**
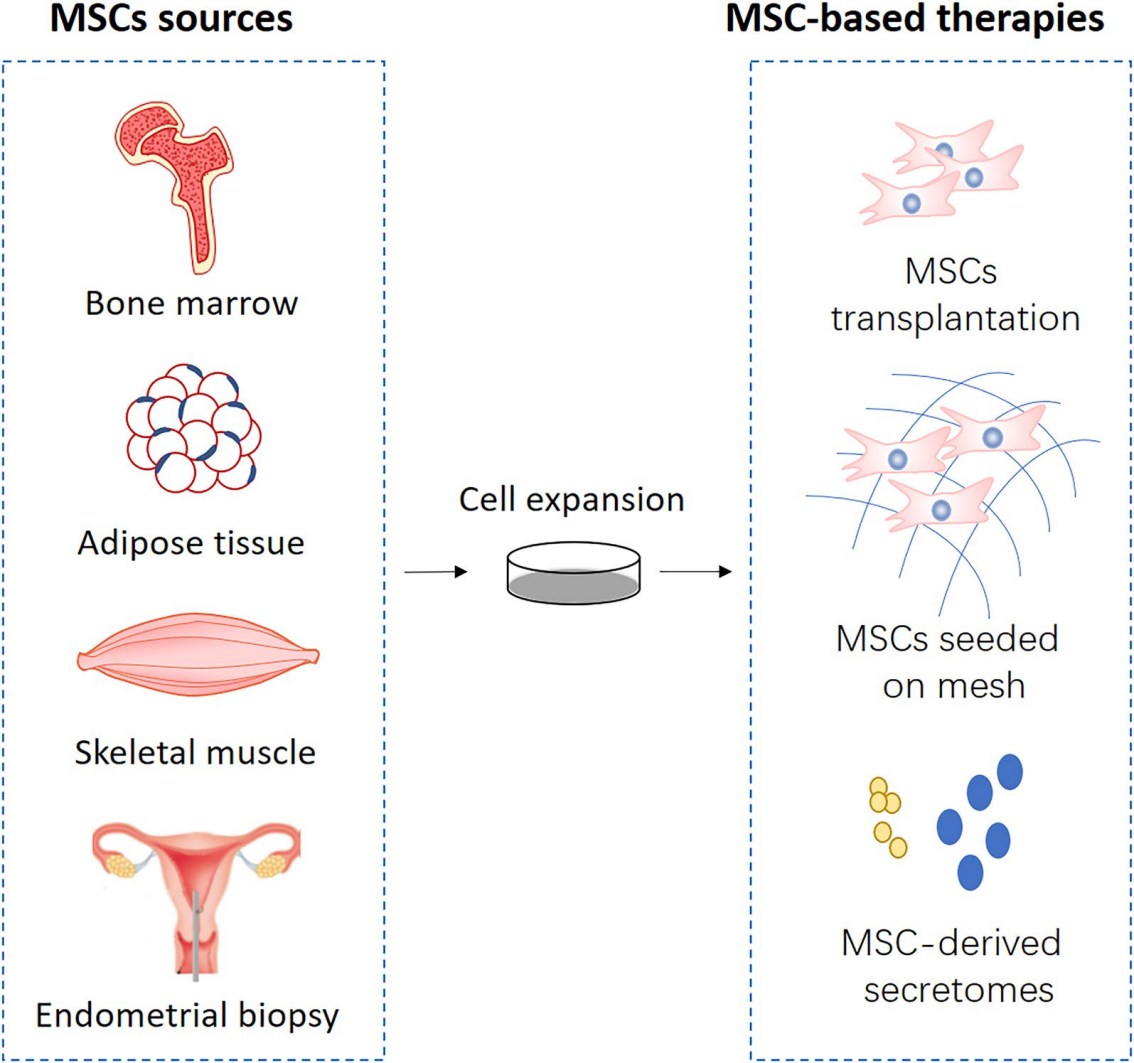
Schematic representation of the different MSCs sources and MSC-based therapies for PFDs

*Adipose*-*derived stem cells (ADSCs)* were first reported being used in an animal model for treating PFDs in 2010 [38]. Then a considerable number of studies focusing on ADSCs-based therapy for PFDs have been carried out both in preclinical and clinical trials. The popularity of ADSCs can be attributed to their biological properties as well as the convenient procurement. According to a clinical study utilizing ADSCs translation to treat SUI, the autologous tissue source was harvested from the patients’ abdomen by liposuction; then ADSCs were isolated from the tissue without culture using the Celution system and finally injected into the urethral sphincter [39]. This treatment can be completed as a single surgical procedure within 3 h. Besides, the injury of isolation site for ADSCs is minimal, and ADSCs are in large quantities in human adipose tissue. Due to these beneficial features for their clinical applications, ADSCs have become a highly investigated cell population in the treatment of PFDs.

*Muscle*-*derived stem cells (MDSCs)* are a population of muscle-resident stem/progenitor cells acquired through muscle tissue biopsies. In fact, there are heterogeneous populations of muscle-resident stem/progenitor cells in skeletal muscle. Apart from the well-known satellite cells that are capable of regenerating muscle fibers, there are groups of non-satellite cells with multilineage potential that are considered belonging to MSCs, which was confirmed by the wide gene expression similar to MSCs [40, 41]. MDSCs have been shown effective and well-tolerated in most clinical studies by urethral injection to treat SUI. MDSCs are also candidate cells of tissue engineering for the therapy of POP, because they are capable of promoting vaginal repair with tissue-engineered scaffolds in rat models [42]. However, the invasive acquisition procedure that often causes significant pain and morbidity is an issue to be solved for the application of MDSCs.

*Endometrial MSCs (eMSCs)* were isolated from human endometrium which is a highly regenerative tissue undergoing more than 400 cycles of growth and shedding during a woman’s reproductive years. Adult human endometrium contains a small quantity of epithelial progenitors and MSCs, which may provide a readily available source of MSCs for cell-based therapies [43, 44]. Recently, several studies combining eMSCs with new biomaterials gained good results in skin wound repair or abdominal hernia animal models [45, 46], demonstrating eMSCs are candidate seeding cells for tissue engineered meshes in the treatment of POMoreover, the convenience of eMSCs acquisition (endometrial biopsies in an office-based procedure) and the discovery that eMSCs can be also isolated from post-menopausal endometrium [47] contribute to their potential clinical use for PFDs.

### More other sources

In additional to the above MSCs, there are some other MSCs sources regarded as candidates for the therapy of PFDs with rare investigations. (1) Umbilical cord-derived MSCs (UC-MSCs) are MSCs derived from various parts of the umbilical cord and particularly from Wharton’s Jelly. Wharton’s Jelly matrix is located close to the vasculature of the cord. MSCs derived from Wharton’s Jelly are called human umbilical cord perivascular cells (HUCPVCs). The similar characteristics between HUCPVCs and BM-MSCs support the applicability of HUCPVCs for cell-based therapies [48]. It has previously been observed that UC-MSCs contributed to the repair of vaginal wall in rats by fabricating a cell-seeded tissue engineering production [49]. (2) Umbilical cord blood-derived MSCs (UCB-MSCs), extracted from human cord blood without invasive procedures, are expected to be useful for cell therapy in regenerative medicine. But the investigation of this cell source in PFDs is rare. The only clinical trial [50] suggested that UCB-MSCs transurethral injection were effective in relieving the symptoms of SUI, which were evaluated by urodynamic study. (3) Placenta-derived MSCs have attracted attention for their immune-modulatory properties and poor immunogenicity, which makes them suitable for allogeneic transplantation. Decidua-derived MSCs, derived from human term decidua, are capable of multilineage differentiation into all three embryonic layers, and they were regarded as a potential source of MSCs for PFDs [51]. (4) Urinary-derived stem cells (USCs) are a subpopulation of cells isolated from human urine, possessing MSC-like features such as clonogenicity, self-renewal, and multipotent differentiation capacity [52, 53]. Moreover, USCs can be obtained noninvasively from human urine specimens. Thus, they are thought to have potential use in genitourinary reconstruction.

MSCs isolated from different tissues exhibit important differences in their availability, characteristics, and regenerative potential. Therefore, the choice of cell source, subsequent isolation, and manipulation techniques depend on the requirements of specific research/clinical applications.

### Roles of MSCs in treatment of PFDs

#### Migration to the site of injury

MSCs have been demonstrated to migrate and situate at the site of injury following infusion, which is also termed as “homing”. MSCs homing is defined as the arrest of MSCs within the vasculature of a tissue followed by transmigration across the endothelium [54]. However, it is unclear if MSCs actively home to tissues using leukocyte-like cell adhesion and transmigration mechanisms or are passively entrapped in small-diameter blood vessels [54]. Although MSCs express many receptors and cell adhesion molecules such as chemokine receptor [55] and integrins [56], the exact mechanisms underlying the migration and homing are not well understood.

**Fig. 2:**
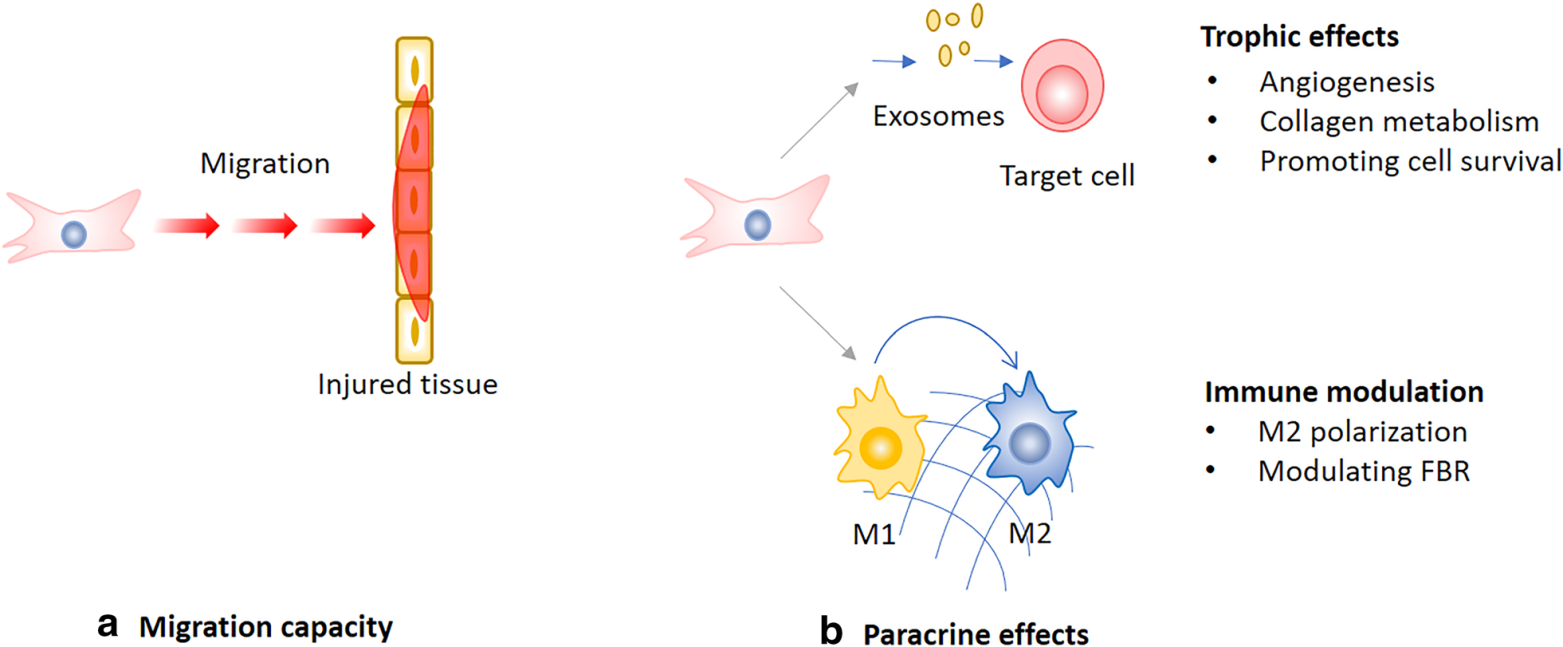
Simplified representation of the potential roles of MSCs in treating PFDs. FBR foreign body response

Current MSC-based therapy for PFDs usually delivers MSCs by periurethral injection. But no matter what injection methods are used, it is impossible to deliver MSCs to the specific site of injury. Considering the connective tissue damages of the pelvic floor are extensive, the homing property of MSCs would play a pivotal role in treating PFDs (Fig. 2a). Cruz et al. found that intravenously injected MSCs distributed to pelvic organs after simulated childbirth injury in a rat model, suggesting that intravenous administration of MSCs could provide an effective route for cell-based therapy [57]. Ben et al. transplanted MSCs systemically or locally to vaginal injury rat model to examine the engraftment, survival, differentiation and angiogenic effect of transplanted MSCs. As a result, both systemic and local MSCs transplantation promoted host angiogenesis, while engraftment after local transplantation was less efficient at all-time points compared to systemic administration, indicating that systemically transplanted MSCs promote tissue repair through homing to the site of injury [58].

#### Paracrine effects

The mechanisms of MSCs therapeutic effects were initially thought as differentiation and cell replacement. However, more and more studies reveal that MSCs exist in vivo for a short time after transplant, in contrast to their long-lasting therapeutic effects. In addition, it is rare to see the injected MSCs engraft into target tissues and differentiate into desired cells. Therefore, many believe that the observed therapeutic effect of MSCs is due to their paracrine effects, also termed as hit-and-run mechanism, which are based on the production of exosomes or secretion of trophic and immunomodulatory factors during the initial days following MSC injection [59] [60]. MSCs secrete a range of proteins/peptides, RNA, hormones, and chemicals by extracellular vesicles such as exosomes or microvesicles [61], and MSC secretions have gained remarkable therapeutic outcomes in preclinical studies. Hence, acellular therapy which harnesses MSC secretions to promote tissue repair is increasingly attractive in regenerative medicine [62]. The paracrine effects of MSCs in the treatment of PFDs can be concluded to trophic effects and immune modulation (Fig. 2b).

##### Promotion of cell survival or trophic effects

MSCs can secrete a broad array of factors that support cell survival, including growth factors, cytokines, and extracellular matrix. Collectively, these secretions have the theoretical capacity to rescue injured cells, reduce tissue damage, and accelerate tissue repair. And this property is exemplified by the natural roles of MSCs as reticular cells that support the hematopoietic stem cell niche and as vascular pericytes that support endothelial cells [63].

In MSC-based therapy for PFDs, paracrine effects of MSCs play an important role in tissue regeneration through promoting the muscular cell survival [64], enhancing the host angiogenesis [58] and modulating the collagen metabolism [65]. Collagen metabolism disorder in connective tissues is one of the well-recognized pathogenic mechanisms of POP, and MSCs may regulate collagen metabolism via paracrine effect to optimize the functional characteristics of fibroblasts. In a preclinical study, the increased collagen I and III productions were observed after a systemic administration of exosomes in the early stage of wound healing, while in the late stage, exosomes might inhibit collagen expression to reduce scar formation [66]. On the contrary, as a novel treatment for fibrotic diseases, MSCs act to reduce TGF β-induced myofibroblasts differentiation and collagen deposition during organ fibrosis [67, 68].

##### Immune modulation

There are a number of publications focusing on the pleotropic effects of MSCs on the immune system. Early studies suggested that MSCs might be immune privileged because MSCs failed to elicit alloreactive lymphocyte proliferative responses [69, 70]. But immune rejections were reported in allogeneic MSCs transplantations. A review by Ankrum et al. [71] provides a thorough discussion on immunogenicity of MSCs and suggests that “MSCs are immune evasive and not immune privileged.” The immunogenic and immunosuppressive properties of MSCs are strongly dependent on context and induced by the inflammatory factors which MSCs are exposed to. Given the diverse immunomodulatory properties of MSCs, MSC-based therapies have been applied in GVHD, sepsis, and some autoimmune diseases. Specifically, MSCs act on both the adaptive and innate immune systems by reducing the activation and proliferation of T and B lymphocytes, suppressing dendritic cell maturation, inhibiting proliferation and cytotoxicity of Natural killer cells, promoting M2 macrophage polarization, and increasing the number of regulatory T cells [72, 73]. The mechanisms of MSCs in mediating these processes are based on their paracrine effects, by secreting inflammatory cytokines such as IL-6, IL-10, TGF-b, PGE2 [74–76].

Herein, the immunomodulatory property of MSCs plays a role in regulating the foreign body response (FBR) when treating POP with the combination of MSCs and biomaterials. FBR is the end-stage response of inflammatory and wound healing processes following medical implantation [77], and ultimately determines rejection or integration of the implanted biomaterial. Synthetic polypropylene meshes, used in pelvic floor reconstructive surgery for POP, have long been blamed for triggering excessive FBR and then causing mesh exposure or erosion. But now, MSCs have been proved to suppress FBR and improve the biocompatibility of meshes in animal models [78, 79] based on the cross-talk between MSCs and immune cells, particularly the macrophages. Therefore, the immunomodulatory property of MSCs may contribute to reducing the surgical complications of POP.

### Preclinical studies of MSCs-based therapy for PFDs

MSCs are highly investigated as a novel therapy for PFDs in a variety of preclinical studies, with different animal models, cell sources, delivery methods and response evaluation systems. MSCs transplantation is the most extensively studied therapeutic strategy in this field, with a long history of therapeutic research for SUI. As the regenerative medicine advances, MSCs-based tissue engineering and MSC-derived exosomes or other secretions emerged as new options for PFDs (Fig. 1).

It is now well established from preclinical studies, that MSCs transplantation is a potential therapeutic strategy for urinary and fecal incontinence (Table 1).

**Table 1:** Selected preclinical studies of MSCs transplantation for PFDs

Author/year	Animal/models	MSC sources	Method of injection/number of cells	Tracking of MSCs	Functional assessments	Main outcomes	Conclusions
Lin et al. 2010 [38]	Rats/VD	Human ADSCs	Urethral or intravenous/1 × 10^6^	Labeled with BrdU and EdU	Conscious cystometry	Urinary voiding function improved, elastin and smooth muscle content increased and MSCs survival for at least 4 weeks.	Transplantation of ADSCs via urethral or intravenous injection was effective in the treatment and/or prevention of SUI in a preclinical setting
Cruz et al. 2012 [57]	Rats/VD	Rat BM-MSCs	Intravenous/2 × 10^6^	Labelled with GFP	/	GFP + MSCs in the pelvic region both 4 and 10 days after VD; the total flux decreased from 4 to 10 days after VD and sham VD	Intravenous administration of MSCs could provide an effective route for cell-based therapy
Sadeghi et al. 2015 [80]	Rats/VD	Human BM-MSCs	Periurethral or intravenous/1 × 10^6^	In situ hybridization; bioluminescence imaging	LPP test	LPP, connective tissue content and vascular density increased in periurethral or intravenous groups; no labeled cells were observed in urethras at 4, 10, and 14 days	Human MSCs restored urinary continence with an immediate and sustained effect in the VD model; MSCs remained at the site of periurethral injection for < 7 days
Menachem- Zidon et al. 2019 [58]	Rats/posterior midline vaginal incision	Rat BM-MSCs	Intravenous or vaginal subepithelial/2 × 10^6^	Labeled with PKH-26 or GFP	/	The epithelial layer healed; systemically transplanted MSCs differentiate into endothelial cells; systemically transplanted MSCs form blood vessel structures	These findings pave the way to further studies of the potential role of MSCs transplantation in improving surgical outcome in women with PFD
Salcedo et al. 2013 [81]	Rats/SP or PNC	Rat BM-MSCs	Intravenous (IV) or intramuscular (IM) into the anal sphincter/2 × 10^6^	Labelled with GFP	Anal pressure test; anal sphincter EMG	Anal sphincter pressure increased in IV and IM groups after SP, but not after PNC; sphincter EMG amplitude also increased in both groups, but frequency only increased in IV group	MSC treatment resulted in significant improvement in anal pressures after SP, suggesting that MSCs could be utilized to facilitate recovery after anal sphincter injury
Salcedo et al. 2014 [82]	Rats/SP	Rat BM-MSCs	IM/5 × 10^5^ or serial IV/5 × 10^5^ daily for 6 consecutive days	Labelled with GFP	Anal pressure test	Both IM and IV MSC treatment after injury caused an increase in anal pressure sustained at 5 weeks	MSCs delivered intravenously and intramuscularly resulted in functional recovery
Kuismanen et al. 2018 [83]	Rats/SP	Human ADSCs	Intramuscular into the external sphincter/3 × 10^5^	Labelled with PMP-50	ARM	ARM pressure was significantly higher in ADSCs treatment groups; No difference in the sphincter muscle continuation between the groups	The ADSCs injection with both saline and Bulkamid is a promising nonsurgical treatment for acute anal sphincter injury
Gautam et al. 2014 [102]	Rabbits/cryoinjured urethral model	Autologous ADSCs	Urethral/2 × 10^6^	Labeled with PKH26	LPP test	LPP of the cell-implanted group was higher compared with control group; implanted PKH26-labeled ADSCs were immunohistochemically positive for myoglobin, SMA, and Pax7 antibodies	Implantation of ADSCs into cryoinjured rabbit urethras promoted the recovery of urethral function

*VD* vaginal dilation, *SP* sphincterotomy, *LLP* leak-point pressure, *PNC* pudendal nerve crush, *ADSCs* adipose-derived stem cells, *BM-MSCs* bone marrow-derived MSCs, *BrdU* 5-bromo-2-deoxyuridine, *EdU* 5-ethynyl-2-deoxyuridine, *GFP* green fluorescent protein, *PMP-50* magnetizable nanoparticles, *EMG* electromyography, *ARM* anorectal manometry, *SAM* smooth muscle actin, *Pax7* a satellite cell marker

Lin et al. [38] first published an investigation applying autologous ADSCs to treat SUI in a rat model. Rats were induced to create an abnormal voiding condition by postpartum vaginal balloon dilation and bilateral ovariectomy. ADSCs were isolated from the rat peri-ovary fat, which was different from the clinical use of the subcutaneous fat because the bilateral ovariectomy was designed to simulate menopause in rats. Then ADSCs were transplanted into the rats via urethral injection or intravenous injection through tail vein. Four weeks later, urinary voiding function was assessed by conscious cystometry and 80% of the control rats had voiding dysfunction, whereas only 33% of the ADSC-treated rats had voiding dysfunction. Normal voiding rats from the ADSC-treated group had significantly higher smooth muscle content and elastin content than the control group or ADSC-treated abnormal voiding rats. These findings suggested that transplanted ADSCs could improve urethral function and the migration of ADSCs toward the injured urethra might be one of the steps through which voiding dysfunction was mitigated. Notably, in this early time research, the labeled MSCs were detectable in the connective tissue till 4 weeks post-transplantation. However, later studies reported that MSCs survived for a short time in spite of producing long-term tissue regenerating effects.

A number of research evidence on the therapeutic effects of MSCs in urinary incontinence support MSCs transplantation as a novel treatment, with restorations in urinary function and structures. However, how long MSCs can survive in situ after an injection has been a debatable question, and different studies reported different survival times of MSCs.

**Fig. 3:**
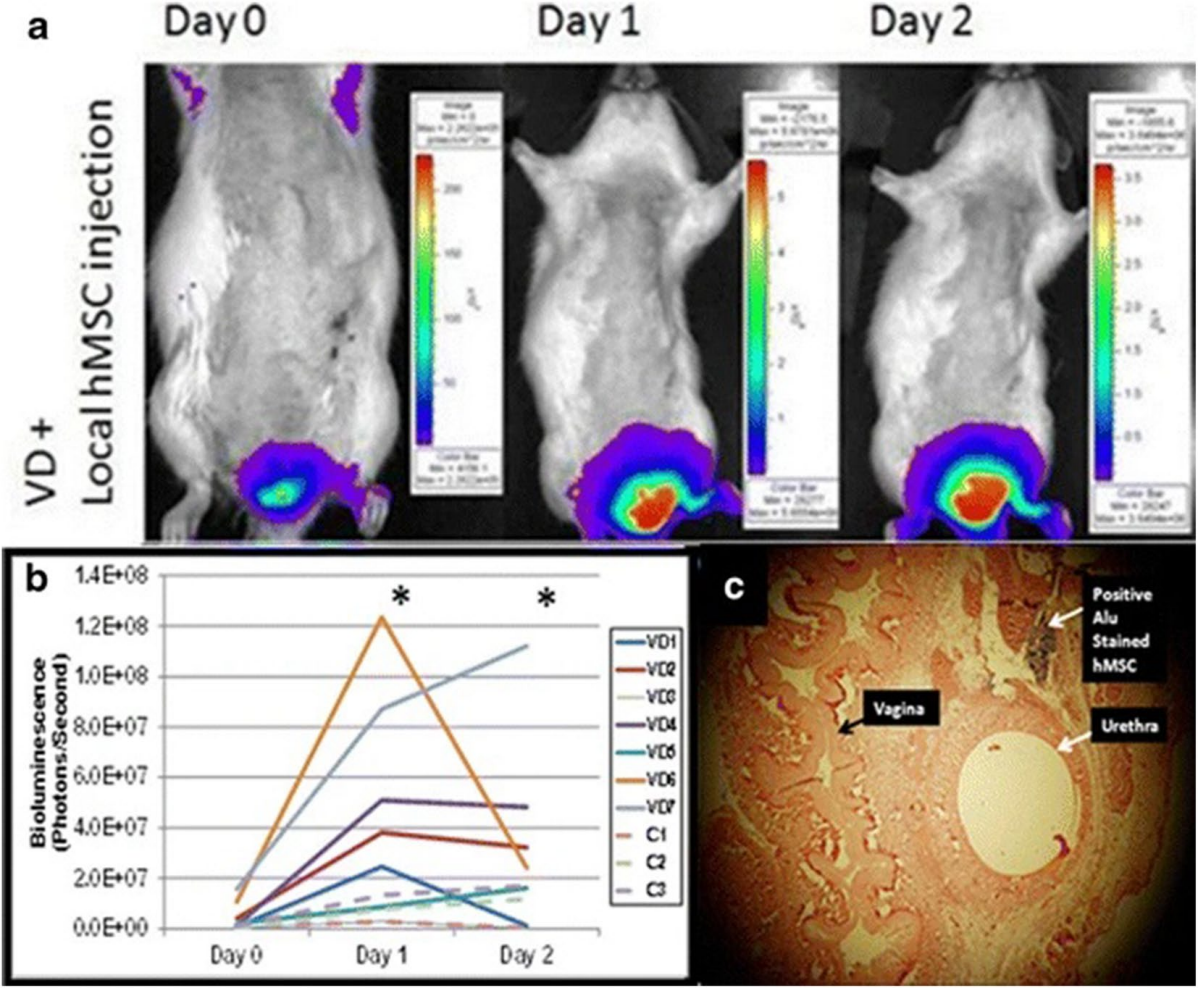
BLI of hMSC localization in VD rats: **a** Representative longitudinal BLI images from a VD rat showing increasing BLI signal on days 1 and 2 following periurethral injection of luciferase-expressing hMSCs. **b** Mean BLI signal in VD animals significantly increased on days 1 and 2 (P < 0.05) in comparison with day 0, suggestive of hMSC recruitment/viability. No significant difference was observed for non-VD rats. **c** Representative urethra 2 h after hMSC injection demonstrates hMSCs: human-specific Alu repeats clearly revealed nuclear staining of hMSCs, whereas no positive Alu signal was found in urethra of imaged animals when BLI signal disappeared. Light microscopy ×40. BLI bioluminescence imaging, hMSC human mesenchymal stem cells, VD vaginal distention. Reprinted with permissions from Sadeghi et al. [80]

Cruz et al. [57] reported a pelvic organ distribution of MSCs after intravenous injection in the rats with vaginal distention (VD). MSCs were transfected and constitutively express a green fluorescent protein (GFP) which could be assessed by fluorescent Imaging. In vivo imaging demonstrated evidence of GFP + MSCs in the pelvic region both 4 and 10 days after VD, but the total flux decreased from 4 to 10 days. Another research that evaluated the potential role of human MSCs in the improvement of urinary continence also explored the fate of injected MSCs [80]. To detect the transplanted MSCs, several approaches were conducted: nuclei were traced using in situ hybridization for human Alu genomic repeats via digoxigenin-labeled DNA probes; in vivo bioluminescence imaging (BLI) was applied to assess MSCs viability and distribution after local periurethral injection in real time. The results showed positively Alu-stained nuclei were observed at 2 h after injection, but were not observed 4, 10, and 14 days after locally and systemically MSCs transplantation. PKH26-labelled cells were also found at 2 and 24 h after injection. BLI signals increased 1 and 2 days after MSCs injection in VD rats, while no significant difference was observed in non-VD rats. (Figure 3). These findings suggested intravenously injected MSCs migrated to the site of injury, which provided an effective route for cell-based therapy to treat SUI; furthermore, MSCs did not promote tissue regeneration in the way of differentiation and replacement considering their rapid disappearance.

Recently, Menachem-Zidon et al. [58] reported a study evaluated the survival, differentiation and angiogenic effects of transplanted MSCs in a vaginal injury rat model established by vaginal incision. Remarkably, the systemically transplanted cells labeled with green florescent protein (GFP) migrated to vaginal injury site and survived for at least 30 days; furthermore, the transplanted cells acquired an endothelial phenotype in vivo, and they were detectable within capillary-like structures. By ruling out the occurrence of fusion between transplanted MSCs and the host endothelial cells, the authors proposed the transplanted MSCs differentiated in situ into endothelial cells. However, the authors didn’t explain why the long-term existence, as well as the endothelial phenotype were only observed in rats with systemically transplanted MSCs, not in those with locally transplanted MSCs. As a matter of fact, whether MSCs are capable of infusion and differentiating into tissue cells, are still controversial. More studies need to be conducted to investigate the fate of transplanted MSCs in vivo.

Apart from the investigations on urinary incontinence, MSCs transplantation has also been studied for fecal incontinence (FI). Salcedo et al. [81] reported that MSCs improved the anal sphincter function in rats with anal incontinence caused by sphincterotomy, but they only measured the anal sphincter pressure 10 days after injury. After that, they further investigated the regenerative effects of MSCs on the injured anal sphincter by comparing anal sphincter pressures following intramuscular and serial intravascular MSCs injection [82]. Anal sphincter pressure increased in both intramuscular and intravascular injection groups, and the increase lasted for 5 weeks. Also, in both MSCs treated groups, less fibrosis and more collagen deposition were found, with the intravascular injection group showing the least scarring. Kuismanen et al. [83] reported similar results of an increase in anal sphincter pressure after MSCs delivery in the same animal model. In addition, a biocompatible carrier, polyacrylamide hydrogel Bulkamid was found to be a suitable carrier for MSCs, because Bulkamid well integrated into the tissue, and a minor foreign body reaction was found in the group receiving Bulkamid with MSCs.

In addition to the efficacy of MSCs treatment, safety of the therapy is a matter of concern. Despite the well-tolerated outcomes of transplanted MSCs in most rat models, there remains a paucity of evidence on the long-term, comprehensive evaluation of the safety. A study tested the dose–effect safety profile of skeletal muscle precursor cells therapy in a sphincter-removed dog model [84]. No adverse effects were found according to the histological pathologic features, blood cell counts, or liver and kidney function markers up to 9 months after cell injection (25–100 million cells per milliliter). This is probably equivalent to around 2–3 years of follow-up in humans.

### MSCs-based tissue engineering for PFDs

Therapies involving tissue engineering, combining MSCs with new materials or meshes were also evaluated in several studies, particularly in the treatment of POP (Table 2). POP is the herniation of pelvic organs into the vagina; hence, the meshes implantation can compensate for inadequate or lack of autologous tissues, to decrease morbidity and to improve long-term efficacy. Many reported MSCs and meshes assisted each other and interacted to improve the final outcomes as a combination. Meshes provide mechanical and structural support for the pelvic tissues as well as offer the cells a scaffold to adhere. Meanwhile, MSCs exhibit immunomodulatory and anti-inflammatory properties to suppress the excessive FBR. Thus, tissue engineering is a new option in the field of pelvic floor repair when soft tissue reinforcement is necessary.

**Table 2:** Selected preclinical studies of MSCs-based tissue engineering for PFDs

Author/year	Animal/models	MSC sources	Materials	Implantation of the constructs	Tracking of MSCs	Main assessments	Main outcomes	Conclusions
Darzi et al. 2018 [86]	Mice/abdominal subcutaneous wound	Human eMSCs	Polyamide/gelatin composite mesh	Implanted into two pockets and sutured to the abdominal fascial layer	Transduced with a mCherry lentivirus	Immunofluorescence; ELISA; qPCR	Higher expression of M2 markers and reduced cytokines in eMSC/mesh; immunomodulatory effects were delayed and weaker in immunocompromised mice	The immune status affected the survival of xenogeneic eMSC which leads to differences in the short-term and long-term macrophage responses to implanted meshes
Ulrich et al. 2014 [78]	Rats/dorsal subcutaneous wound	Human eMSCs	Polyamide/gelatin mesh	Implanted into a subcutaneous pocket; two meshes inserted for each rat	Labeled with DiO	Histological analysis; immunofluorescence; uniaxial tensiometry	MSCs detected on the mesh up to 14 days; Meshes with MSCs promoted neovascularization and reduced leukocyte infiltration	Seeding with eMSC exerted an anti-inflammatory effect and promoted wound repair, and produced mesh with greater extensibility
Ding et al. 2018 [49]	Rats/posterior vaginal wall incision	Human UC-MSCs	PP mesh	Implanted into vaginal wall next to the rectovaginal fascia	Marked with GFP or RFP	Macroscopic evaluation; fluorescence microscopy; histological analysis	No difference in fibrotic remodeling and inflammatory cells number; a better vascularization in cell-seeded mesh and a thicker layer covered the cell-seeded scaffold	UC-MSCs with differentiated smooth muscle cells might have a potential role in fascia tissue engineering to repair POP in the future
Edwards et al. 2015 [85]	Rats/dorsal subcutaneous wound	Human eMSCs	Polyamide/gelatin mesh	Implanted into a subcutaneous pocket and secured to the muscle layer	/	Uniaxial biomechanical analysis; scanning electron microscopy	Cell-seeded scaffolds were significantly less stiff than non-cell-seeded scaffolds; Collagen growth and organization were enhanced in the long-term in cell-seeded scaffolds	Results suggest that neo-tissue formation and remodelling may be enhanced through seeding scaffolds with eMSCs
Paul et al. 2019 [46]	Rats/abdominal subcutaneous wound	Human eMSCs	3D printed PCL mesh	Implanted into a subcutaneous pocket with cell side facing abdominal wall	Transduced with a mCherry lentivirus	Scanning electron microscope; Atomic Force Microscopy; Fourier Transform Infrared Spectroscopy; histological analysis	eMSC printed on MES constructs promoted tissue integration, eMSC retention and an anti-inflammatory M2 macrophage phenotyp	eMSC bioprinting onto an MES mesh to produce a CAD-specific potentially surgical grade tissue engineered construct for possible urogynecological applications such as POP

*eMSCs* endometrial MSCs, *UC-MSCs* umbilical cord-derived MSCs, *PP* polypropylene, *PCL* poly caprolactone, *RFP* red fluorescent protein, *ELISA* enzyme-linked immunosorbent assay, *qPCR* quantitative polymerase chain reaction

**Fig. 4:**
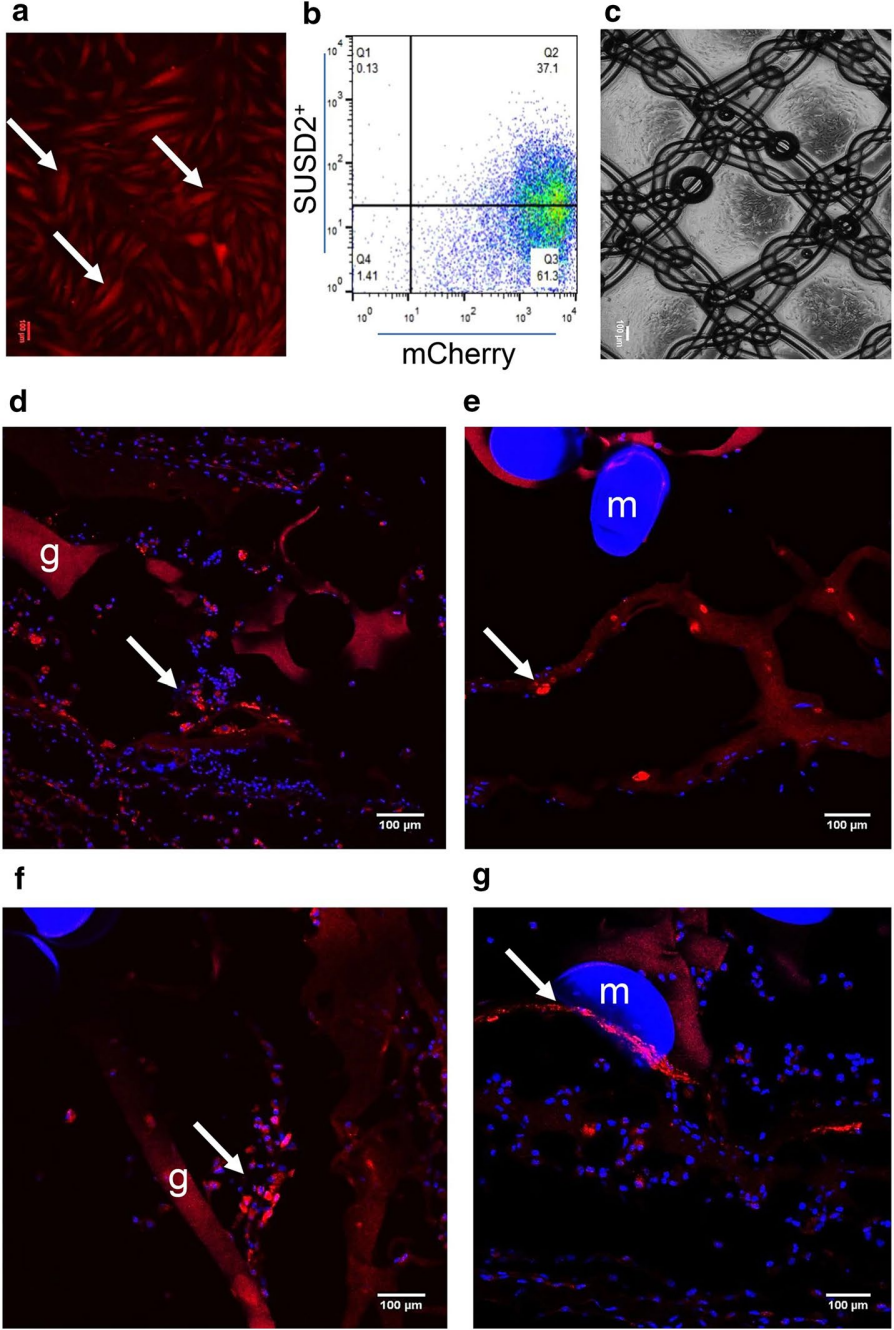
eMSC transduction and survival of eMSC on PA + G mesh in NSG mice. **a** cultured mCherry transduced eMSC showing red fluorescence, **b** more than 95% of transduced and cultured eMSC were mCherry + by flow cytometry and about 40% of this population were SUSD2+. Representative trace of n = 6 patient samples, **c** PA + G mesh seeded and cultured with eMSC. **d, e** mCherry + eMSC were observed 3 and **f, g** 7 days post-implantation around the mesh filaments in immunocompromised NSG mice. Arrows, representative mCherry + eMSC; m, mesh filament; g, gelatin. Scale Bars 100 µm. Reprinted with permissions from Darzi et al. [86]

In recent years, there has been an increasing interest in applying eMSCs to tissue engineering therapy for pelvic floor repair [46, 78, 85] and the eMSCs have exhibited an excellent modulatory property to the extra cellular matrix remodeling and the inflammatory reactions, but the mechanism remains unclear. A study [86] characterized some of the immunomodulatory properties of eMSCs in vivo to understand the immunoregulatory mechanism of eMSCs on macrophages. The authors implanted polyamide/gelatin composite mesh seeding with mCherry lentivirus-labelled eMSCs to the abdominal subcutaneous wounds in C57BL6 immunocompetent and NSG immunocompromised mice. Dual color immunofluorescence staining was performed to quantify M1 and M2 macrophages. Results showed that eMSCs were detected around the mesh in NSG mice but not in C57BL6 mice on 3 and 7 days after implantation (Fig. 4). Both in NSG and C57BL6 mice models, the M2/M1 ratio was higher and the expression of M2 macrophage markers increased in eMSC/mesh compared to mesh control. Also, the inflammatory cytokine IL-1β and TNF-α reduced in eMSC/mesh compared to mesh control. These immunomodulatory effects were delayed and weaker in NSG mice compared to C57BL6 mice. In sum, the eMSC-modulated macrophage responses to synthetic meshes differed in immunocompetent and immunocompromised mice. It is apparent that the eMSC exerted these immunomodulatory effects via a paracrine mechanism, since eMSCs disappeared rapidly after implantation while the anti-inflammatory effect lasted to 30 days.

### MSCs-derived secretome for PFDs

The spectrum of regulatory and trophic factors secreted by MSCs, including exosomes, cytokines, and chemokines, is broadly defined as the MSC secretome. With an awareness shift of MSCs therapeutic effects from differentiation to secretion, many studies harnessed MSC secretome to treat various diseases [30, 87]. This new MSCs-based therapy, known as acellular therapy, can provide therapeutic benefits without the need to transplant living cells, making the process easier to be standardized and reducing cell transplant related risks. Several attempts have been made to investigate the therapeutic effects of MSC secretome in PFDs (Table 3), by utilizing the concentrated conditioned media (CCM) of MSCs [64, 88] or the exosomes derived from MSCs [89] [90].

**Table 3:** Selected preclinical studies of MSCs-derived secretome for PFDs

Author/year	Animal/models	MSC sources	Method of injection	Characterization of exosomes	Functional assessments	Main outcomes	Conclusions
Dissaranan et al. 2014 [64]	Rats/VD	Rat BM-MSCs	Periurethral or intravenous	Using CCM not exosomes	LPP test; external urethral sphincter electromyography	LPP, elastin fibers and urethral smooth muscle increased in rats treated with MSCs or CCM	MSCs homed to the urethra and vagina and facilitated recovery of continence most likely via secretion of paracrine factors
Liu et al. 2018 [65]	In vitro experiments, vaginal fibroblasts from women with SUI	Human ADSCs	/	/	/	The expression of type I collagen, TIMP-1 and TIMP-3 increased, whereas the expression of MMP-1 and MMP-2 decreased	Exosomes increased type I collagen contents by increasing collagen synthesis and decreasing collagen degradation
Ni et al. 2018 [89]	Rats/PNT and VD	Human ADSCs	Periurethral	Dynamic light scattering; scanning electron microscopy; identification of surface markers; proteomic analysis	Cystometrography; LPP test	Exosomes enhanced the proliferation of muscular and Schwann cell in vitro; the bladder capacity, LPP, striated muscle fibers, and nerve fibers increased both in ADSCs-treated and exosomes-treated rats	Local injection of ADSCs-derived exosomes improved functional and histological recovery after SUI
Wu et al. 2019 [90]	Rats/VD	Human USCs	Local injection around the pubococcygeus muscle	Identification of surface markers; assessments of size distribution and concentration	Maximum bladder volume; abdominal LPP test	Exosomes improved the urodynamic parameters; promoted the pubococcygeus muscle recovery, satellite cells activation, proliferation and differentiation; promoted ERK phosphorylation	USCs-derived exosomes played roles in myogenic satellite cells differentiation into myoblasts and in SUI cell-free treatment

*VD* vaginal dilation, *SUI* stress urinary incontinence, *ADSCs* adipose-derived stem cells, *BM-MSCs* bone marrow-derived MSCs, *PNT* pudendal nerve transection, *USCs* urinary-derived stem cells, *CCM* concentrated conditioned media, *LPP* leak-point pressure, *TIMP* tissue inhibitor of metalloproteinases *MMP* matrix metalloproteinase

An in vivo study from Liu et al. [65] evaluated the effects of human MSC-derived exosomes on collagen metabolism in cultured fibroblasts from postmenopausal women with or without SUI. Exosomes were prepared by ultracentrifugation of MSCs conditioned medium and were confirmed by transmission electron microscopy and western blot analysis. After 6-h culture, the expression of type I collagen, TIMP-1 and TIMP-3 increased, whereas the expression of MMP-1 and MMP-2 decreased in vaginal fibroblasts treated with exosomes. The results suggested that exosomes increased type I collagen contents by increasing collagen synthesis and decreasing collagen degradation in vaginal fibroblasts from women with SUI.

In another research from Ni et al. [89], the therapeutic potential of human ADSCs-derived exosomes in SUI was studied in vivo and in vitro. The methods of exosomes isolation and characterization were similar to Liu’s research. SUI model was established by pudendal nerve transection (PNT) and vaginal dilation (VD) in female rats and rats were peripheral urethral injected with ADSCs or ADSCs-derived exosomes. In-vitro results showed exosomes could enhance the growth of skeletal muscle and Schwann cell lines in a dose dependent manner. In-vivo experiments illustrated that rats of the exosome group had higher bladder capacity and leak point pressure (LPP), and had more striated muscle fibers and peripheral nerve fibers in the urethra than rats of control grouTo explain how the exosomes benefit the recovery of SUI, the authors performed proteomic analysis and found ADSC-derived exosomes contained a variety of proteins related to skeletal muscle and nerve regeneration, but the precise mechanisms underlying the phenomenon are still unknown.

Similarly, a recent study [90] reported the therapeutic effects of MSCs-derived exosomes on SUI in a rat model, and illustrated the therapeutic effects in skeletal muscle regeneration were related to the phosphorylation of extracellular-regulated protein kinases (ERK) in satellite cells (SCs). Histological analysis showed fibrosis and muscle morphology were close to normal in pubococcygeus muscle after 8 weeks of exosomes injection. Moreover, after exosomes injection, the activation, proliferation, and differentiation of SCs were promoted; the phosphorylation of ERK was enhanced; nevertheless, the myogenic effect of exosomes almost disappeared in the presence of ERK inhibitor.

In summary, MSC-based therapies for PFDs have been tested in small animal models and have significantly improved PFDs symptoms. In these studies, rat animal models were mostly established by sphincter injury or vaginal distension and less frequently by pudendal nerve injury or vaginal incision. Such animal models are unsatisfactory because they partially mimic the disease mechanism or symptoms, and there is no gold standard animal model for PFDs. Thus, more other improved animal models should be utilized in future investigation. As for the transplanted methods, periurethral injection was used in all preclinical trials, while intravenous injection was used in some studies to classify the migration property of MSCs and to compare these two injection methods. In addition, it needs to be noticed that some of the preclinical studies reported conflicting results of the survival time and in vivo differentiation potential of the injected MSCs. These discrepancies in research outcomes may be explained by the fact that minor differences in the cell sources, culture conditions and cell dosages between these studies can profoundly affect the functions of the injected MSCs as well as the effectiveness of the therapy. Therefore, future research should focus on determining a standardized protocol of MSC-based therapy for PFDs.

### Clinical trials of MSCs-based therapy for PFDs

The effectiveness of MSCs transplantation therapy in PFDs has been demonstrated in pre-clinical studies, leading to its evaluation in several clinical studies. To date, only a few small clinical studies focusing on MSCs therapy for SUI and FI have been reported. Most of them are phase I/II clinical trials, with a small number of subjects (Table 4).

**Table 4:** Selected clinical trials of MSCs-based therapy for PFDs

Author/year	Conditions	Number of patients	MSC sources	Harvesting tissue	Method of injection/number of cells	Follow-up	Clinical evaluation	Clinical outcomes	Adverse events
Kuismanen et al. 2014 [96]	SUI	5	Autologous ADSCs	Abdominal subcutaneous adipose tissue	Transurethral/2.5 × 10^6^to 8.5 × 10^6^	12 months	Cough test; 24-h pad test; questionnaires	The cough test was negative for three patients; questionnaire scores improved	Small hematomas
Carr et al. 2013 [94]	SUI (not improved with conservative therapy)	38	AMDCs	Quadriceps femoris	Intrasphincter/low doses (1, 2, 4, 8, 16 × 10^6^) or high doses (32, 64, 128 × 10^6^)	18 months	3-day voiding diaries; 24-hour pad test; questionnaires	The mean pad weight and mean leakage frequency reduced; better outcomes in high dose vs the low dose group; questionnaire scores improved	Urinary tract infection
Peters et al. 2014 [95]	SUI (refractory to prior treatment)	80	AMDCs	Quadriceps femoris	Intrasphincter/10, 50, 100, 200 × 10^6^	12 months	3-day voiding diaries; 24-hour pad tests; questionnaires	The diary leakage frequency reduced within 1 to 3 months; only patients who received 200 × 10^6^ cells had a reduction in mean pad weight; questionnaire scores improved	Biopsy related adverse
Sèbe et al. 2010 [91]	SUI with fixed urethra (after previous failed surgical management)	12	AMDCs	Deltoid muscle	Endourethral/1 × 10^7^, 2.5 × 10^7^, and 5 × 10^7^	12 months	7-days bladder diary; 1-h pad test; questionnaires	Pad tests were negative for three patients; two patients were slightly worsened; questionnaire scores improved	Three cases of urinary tract infection
Stangel-Wojcikiewicz et al. 2014 [92]	SUI of degrees I or II	16	AMDCs	Deltoid muscle	Transurethral/0.6 × 10^6^to 5 × 10^6^	2 years	Cough LLP; Valsalva LLP; urodynamic studies; questionnaires	Continent (50%), some improvement (25%), no improvement (25%)	No severe adverse effects
Stangel-Wojcikiewicz et al. 2016 [93]	SUI of degrees I or II	16	AMDCs	Deltoid muscle	Transurethral/0.6 × 10^6^ to 25 × 10^6^	4 years	I-QOL questionnaire	The total I-QOL score increased from 49 before therapy to 77 2 years post-operation	No severe adverse effects
Arjmand et al. 2017 [97]	SUI (6 combined with POP)	10	Autologous ADSCs	Abdominal subcutaneous adipose tissue	Periurethral/1.18 × 10^7^	24 weeks	24 h voiding diary; 24-h pad test; urodynamics and uroflow studies; ICIQ questionnaire	Pad test weight and ICIQ scores were improved; maximum flow rate improved.	one case of slight voiding difficulty
Frudinger et al. 2018 [98]	FI	39 (34 females and 5 males)	AMDCs	Musculus pectoralis major	Injected into the external anal sphincter/15 × 10^7^	12 months	Weekly incontinence episodes (WIE); Incontinence diary; Wexner scores; anorectal manometry tests	In 79.5% of patients, the WIE frequency had decreased by at least 50%.	No severe adverse effects
Sarveazad et al. 2017 [99]	FI (after sphincteroplasty)	20	Autologous ADSCs	Superficial abdominal fat tissue	Injected into the external anal sphincter/6 × 10^7^	8 weeks	Wexner scores; endorectal sonography; electromyography (EMG)	Ratio of the area occupied by the muscle to total area of the lesion increased; no difference in Wexner scores between the groups	one case of erythema at the site of surgery
Jankowski et al. 2018 [100]	SUI	143	AMDCs	Vastus lateralis	Intrasphincter/15 × 10^7^	2 years	Incontinence episode frequency (IEF); 24-h or in-office pad weight tests; questionnaires	Responder rates for the endpoint were similar between groups; with the stringent endpoint, a relationship was between IQOL scores and the responder rates	No severe adverse effects

*SUI* stress urinary incontinence, *FI* fecal incontinence, *AMDCs* autologous muscle derived cells, *ADSCs* derived MSCs, *ADSCs* adipose-derived stem cells, *LLP* leak-point pressure, *I-QOL* incontinence quality of life scale, *ICIQ* international consultation on incontinence questionnaire

Clinical trials investigating the treatment of SUI using autologous muscle derived cells (AMDCs) have shown the treatment was well-tolerated and, in some subjects, effective. Notably, AMDCs used in these trials are not a simple cell population, but a mixture of fibroblasts and myogenic cells which were identified through skeletal muscle marker expression. Sèbe et al. [91] evaluated the safety and efficacy for the intrasphincteric injections of AMDCs in women with severe SUI. There were no severe adverse effects, and three cases of urinary tract infection were reported according to the positive urine culture. Wojcikiewicz et al. [92] published a 2-year follow-up investigation on AMDCs in SUI, with a 75% success rate according to the stress test evaluations and questionnaire scores. Then the authors conducted assessments based on validated questionnaires at 2 and 4 years after the cell therapy, and the autologous cell therapy significantly improves quality of life as well as psychological condition in those patients [93]. To identify the optimal cell dose for cell therapy, Carr et al. [94] compared different intrasphincteric injection doses (varying from 1 × 10^6^ to 128 × 10^6^) of AMDCs, and better clinical outcomes were observed in patients with higher doses. Using the same method of cell isolation and urinary incontinence evaluations, Peters et al. [95] researched with an expanded sample and determined 4 different cell doses were effective and tolerated in SUI patients.

Similarly, adipose derived MSCs also have been used for the treatment of SUI in clinical trials. A pilot study conducted by Kuismanen et al. [96] revealed that 3 of 5 patients displayed a negative cough test and questionnaires showed subjective improvement in all patients at 1 year after the injection of ADSCs with collagen gel. Arjmand et al. [97] transplanted ADSCs into the periurethral region of 10 patients and presented a short-term outcome of the treatment. Urinary incontinence was significantly improved, and no complication was reported except one patient experienced slight voiding difficulty.

In addition to SUI, fecal incontinence, as another condition in PFDs, has been investigated with MSCs transplantation in clinical research. Frudinger et al. [98] injected AMDCs into the external anal sphincter in 39 (34 females and 5 males) patients and found the weekly incontinence episodes frequency reduced, with a high degree of patients’ satisfaction. However, despite of the positive outcome of symptoms, the authors didn’t observe physiological changes by anorectal manometry or ultrasound. Sarveazad et al. [99] conducted a randomized double-blind clinical trial to evaluate the efficacy of ADSCs transplantation in 20 female patients with sphincter defects. There was no difference of the Wexner score that was used to check muscle function between cell group and control group; the endorectal sonography and electromyography results showed an increase of muscle tissue at the repair site, but the results were not confirmed by biopsy or magnetic resonance imaging.

**Fig. 5:**
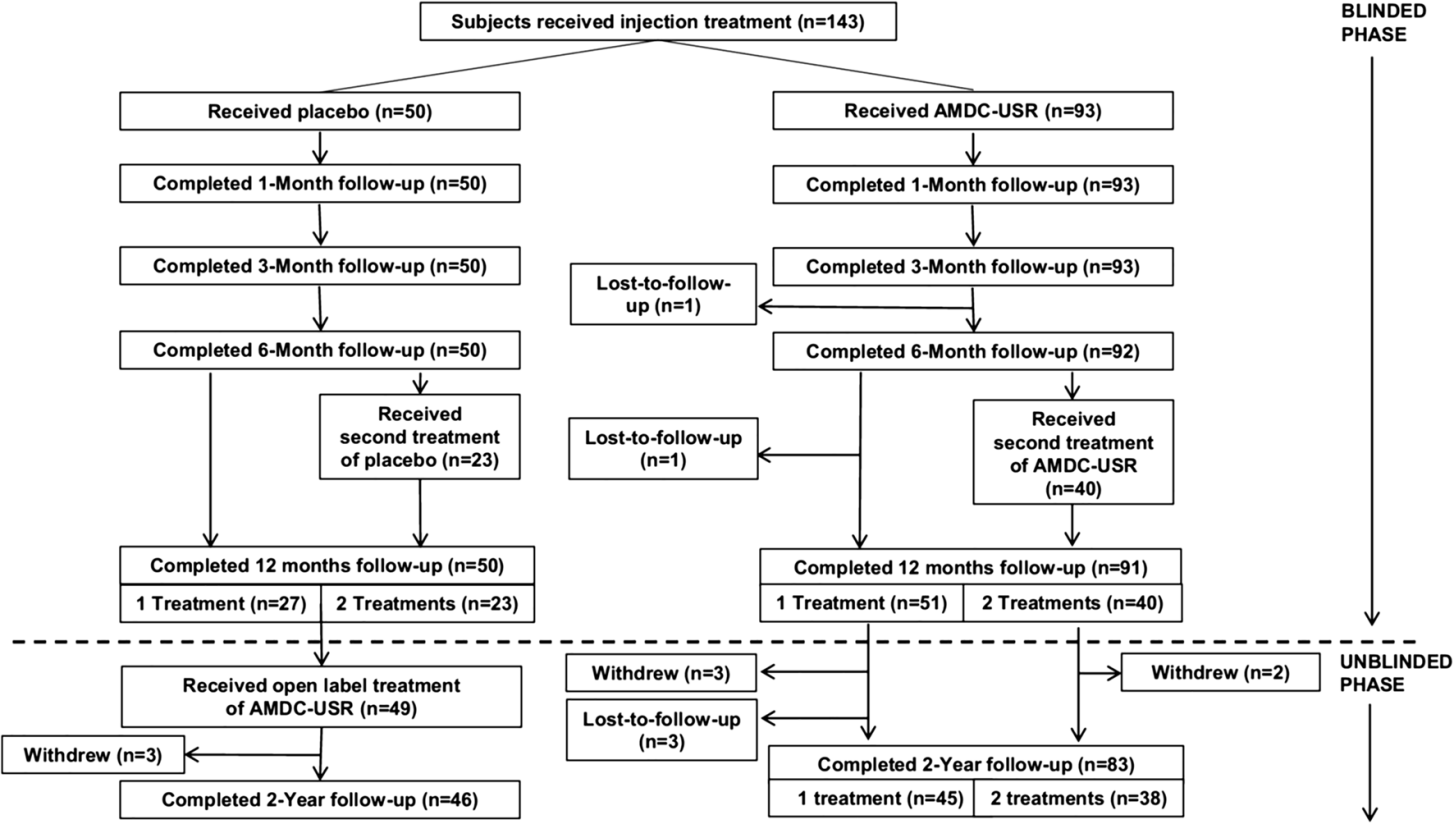
Subject disposition. AMDC-USR autologous muscle derived cells for urinary sphincter repair; n number of subjects. Reprinted with permissions from Ron J. Jankowski et al. (2018)

In 2018, a double-blind, randomized, placebo-controlled clinical trial (Fig. 5) [100] was published to evaluate the safety and efficacy of AMDCs in female subjects with SUI. The primary outcome data included stress incontinence episode frequency (IEF), 24-h in-home pad tests, in-office pad tests and Incontinence Quality of Life Scale (IQOL). The responder rates over 12 months for the composite endpoints that included at least 50% reductions in stress IEF, or 24-h pad weight test, or in-office pad weight test compared with baseline were similar between placebo and AMDCs groups, suggesting a high placebo responder rate. Then by using the more stringent endpoints that included at least 75% reduction in stress IEF or at most 1 stress incontinence episode reported over 3 days, a greater reduction of the responder rate was observed in the placebo group compared with AMDCs group, but the difference was not statistically significant. Besides, the improvements in median IQOL scores were statistically significant higher for subjects who indicated a response to therapy compared with subjects who did not, according to the stringent endpoint. This post hoc analysis of the relationship between reduction in stress IEF and change in IQOL scores substantiated stress IEF as a clinically meaningful endpoint that may be used to better assess efficacy in future studies. In conclusion, although the interim analysis revealed an unexpectedly high placebo response rate and that resulted in a decrease in the evaluable sample size, the clinical trial is the largest clinical study to date in investigations on cell therapy for SUI and demonstrated AMDCs transplantation as a well-tolerated treatment for SUI. Besides, a large placebo effect is common among the clinical trials of cell therapy, making the efficacy of this therapy difficult to be evaluated in clinical trials. In spite of its limitations, the study certainly adds to our understanding of cell therapy for SUI and provides references on the study design for the future trials.

The use of MSCs seems to be a feasible and safe strategy with therapeutic effects for patients with SUI. However, as a result of the heterogeneities in preclinical and clinical trials, the standardized protocol of MSC-based therapy in SUI is still under investigation. As yet, there has been no optimal choice of cell types, cell doses, and cell injection methods for the investigation of autologous MSCs in the therapy of PFDs. Moreover, unlike in animal studies, it is difficult to trace the injected cell fate in clinical trials. This indicates a need to find appropriate avenues for human subjects to detect the cell distribution, proliferation, and differentiation of in vivo MSCs. In addition, the therapeutic efficacy is not as good as expected in clinical trials; some patients were not responsible to the therapy. The reason for the low efficacy of cell therapy in patients with SUI is not clear but it may have something to do with the insufficient precision of cell delivery. A preclinical study [101] aiming to determine the injection accuracy rate both with transurethral and periurethral route was performed in female goats. Although majority of cell depots were administrated accurately into the urethral wall, the precise delivery of cells into external urethral sphincter is limited regardless of injection method.

In conclusion, whilst these current clinical trials did not confirm the effectiveness and safety of MSCs transplantation, they did partially substantiate MSCs transplantation as a promising alternative therapy for urinary and fecal incontinence by improving the urethral or anal sphincter function. Notwithstanding the relatively limited samples in these studies, they offer valuable insights into MSCs application in disease therapy and provide references for the future studies. Further clinical trials, with larger sample size, unified MSCs handling methods, and incorporation of a placebo control group, could shed more light on investigating MSC-based therapy for PFDs.

## Conclusion

Currently, as the most commonly used cells in regenerative medicine, MSCs are highly investigated for PFDs owing to their rich sources, convenient acquisition, and pleiotropic effects. Existing preclinical research recognizes that MSCs exhibit a strong capacity for tissue regeneration and immune modulation by delivery of MSCs or MSCs secretions or MSCs seeded meshes. Furthermore, the therapeutic effects of MSCs transplantation for PFDs have also been underscored by several clinical trials. Whilst the mechanisms that underpin the therapeutic effects are not fully understood, in current studies, MSCs are considered as acting by secreting a large array of bioactive molecules to optimize the target cell functions and to regulate the immune responses.

These findings provide the following insights for future research. Firstly, basic work is needed to fully understand the nature of MSCs, which includes their origins, biomarkers, and biological properties. Secondly, a standardized protocol of MSC-based therapy should be established since the heterogeneous procedures for cell isolation, cultivation, and transplantation would pose a risk to the safety of MSCs clinical applications. Last but not least, future randomized controlled trials with large sample size should be carried out to evaluate the efficacy and safety of MSC-based therapy for PFDs.

Besides, as MSCs-derived extracellular vesicle such as exosome is fast becoming a key instrument in tissue repair and regeneration, acellular therapy is now regarded as a promising strategy for PFDs. Its therapeutic effects have been observed in animal models, and there’s no worry about the safety issues related to cell transplantation. At present, there are few clinical trials of acellular therapy that are conducted to validate the effectiveness in patients with PFDs. Therefore, the development of acellular treatments offers a significant opportunity in the process of seeking new therapies for PFDs.

Undoubtedly, MSCs possess the therapeutic potential for PFDs as well as many other diseases, but MSC-based therapy for PFDs is still at an experimental stage. Moving forward, more investigations need to be conducted to improve the efficacy and ensure the safety of MSCs-based therapy before it is applied to the clinical treatment of PFDs.

## Data Availability

Not applicable.

## Abbreviations

MSCs: Mesenchymal stem cells
PFDs: Pelvic floor disorders
UI: Urinary incontinence
SUI: Stress urinary incontinence
FI: Fecal incontinence
POP: Pelvic organ prolapse
GVHD: Graft-versus-host disease
BM-MSCs: Bone marrow-derived MSCs
ADSCs: Adipose-derived stem cells
eMSCs: Endometrial MSCs
MDSCs: Muscle-derived stem cells
UC-MSCs: Umbilical cord-derived MSCs
HUCPVCs: Human umbilical cord perivascular cells
UCB-MSCs: Umbilical cord blood-derived MSCs
USCs: Urinary-derived stem cells
HLA-II: Human leukocyte antigen II
IL: Interleukin
TGF-b: Transforming growth factor beta
PGE: Prostaglandins E
FBR: Foreign body response
GFP: Green fluorescent protein
BLI: Bioluminescence imaging
CCM: Concentrated conditioned media
MMP: Matrix metalloproteinase
TIMP: Tissue inhibitor of metalloproteinases
PNT: Pudendal nerve transection
PNC: Pudendal nerve crush
VD: Vaginal dilation
SP: Sphincterotomy
AMDCs: Autologous muscle derived cells
ERK: Extracellular-regulated protein kinases
SCs: Satellite cells
IEF: Incontinence episode frequency
IQOL: Incontinence quality of life scale
